# Efficacy of Dual-Light Photodynamic Therapy on Oral Health in Adolescents Undergoing Fixed Orthodontic Treatment: A Randomized, Assessor-Blinded and Controlled Trial

**DOI:** 10.3390/dj14090564

**Published:** 2026-09-04

**Authors:** Katja Hashemi Elses, Tommi Pätilä, Ann-Marie Roos Jansåker

**Affiliations:** 1 Department of Periodontology, Faculty of Odontology, Malmö University, SE-205 06 Malmö, Sweden; katja.hashemi-elses@mau.se (K.H.E.);; 2 Ortodonti Lund, Borgska Villans Specialisttandvård, Hedvig Möllers Gatan 17, SE-223 55 Lund, Sweden; 3 Department of Congenital Heart Surgery and Organ Transplantation, New Children’s Hospital, University of Helsinki, 00290 Helsinki, Finland

**Keywords:** antibacterial photodynamic therapy, dual-light aPDT, indocyanine green, oral hygiene, fixed orthodontic treatment, adolescents, plaque control, gingival inflammation, bleeding on probing

## Abstract

**Background/Objectives**: Patients with fixed orthodontic appliances are at increased risk of plaque accumulation, gingivitis, and caries because brackets and bands hinder mechanical hygiene. This study evaluated whether regular home use of dual-light antibacterial photodynamic therapy (aPDT) supports oral hygiene during fixed orthodontic treatment in adolescents. **Methods**: In a single-center, randomized, assessor-blinded trial, forty-one adolescents starting fixed orthodontic treatment were randomised to a Control group (standard hygiene) or a Test group (standard hygiene plus home-use dual-light aPDT). Visible Plaque Index (VPI%), Bleeding on Probing (BOP%), and Orthodontic Plaque Index (OPI) were assessed at baseline and 12 weeks by an assessor blinded to allocation. After two protocol-driven exclusions, 39 participants (20 Test group, 19 Control group) were analysed. Site-level proportions were compared using the chi-square test. **Results**: VPI decreased in both groups, with a larger mean within-group reduction in the Test group (−7.0%) than in the Control group (−3.7%); exploratory site-level analysis showed a greater reduction in plaque-positive sites in the Test group at 12 weeks (*p* < 0.001). BOP increased in both groups but to a smaller extent in the Test group (mean +2.4% versus +5.7%), and a higher proportion of Test-group participants maintained BOP ≤ 10% (85% versus 58%); bleeding-positive sites were lower in the Test group at 12 weeks (*p* < 0.001). The Test group also showed a flatter distribution of OPI scores around orthodontic brackets. No device- or protocol-related adverse events were reported. **Conclusions**: In adolescents undergoing fixed orthodontic treatment, regular home use of dual-light aPDT was safe and associated with better site-level plaque and bleeding control, with directional but non-significant trends at the participant level. The findings support further evaluation of dual-light aPDT as an adjunct to mechanical hygiene during this vulnerable treatment period.

## 1. Introduction

Orthodontic therapy is one of the most common dental treatments for children and adolescents [1]. Patients fitted with fixed orthodontic appliances have an increased risk of caries, gingivitis, and periodontal disease, partly mediated by changes in the oral microbiota [2,3,4,5].

Fixed appliances also compromise patients’ ability to maintain optimal hygiene [5], and adolescents in particular show fluctuating motivation and technique [6]. Insufficient hygiene leads to substantial plaque build-up around brackets and bands [7,8,9]. Beyond professional cleaning, daily self-performed hygiene remains the cornerstone of biofilm control [10,11,12,13], so patients undergoing fixed orthodontic treatment must be carefully instructed in plaque removal [6,10,14].

While chlorhexidine-based mouth rinses have long been considered effective, their use is limited by common side effects, including mucosal irritation, tooth staining, and taste disturbances [15,16,17]. A range of newer tools—combination mouthrinses, mineral-enriched toothpastes, novel flossing devices, and powered toothbrushes—have been introduced to enhance plaque control [18]. Antibacterial photodynamic therapy (aPDT) has emerged as a promising adjunct for improving oral hygiene [19,20]. Until recently, however, its use has been largely restricted to in-clinic applications, because the need for specialised equipment and professional administration has limited the protocols available and made regular aPDT inaccessible for most patients [21,22].

A home-use dual-light aPDT system, combining an indocyanine green photosensitiser with 405 nm blue and 810 nm near-infrared light, offers a non-invasive way to target plaque biofilms [23] and has been shown to be safe and effective for daily home use [24,25,26].

The study was prospectively registered at ClinicalTrials.gov on 11 April 2023, with the identifier: NCT05825742, https://clinicaltrials.gov/study/NCT05825742 (accessed 17 June 2026).

## 2. Materials and Methods

This study evaluated whether a home-use dual-light aPDT device, used as an adjunct to standard hygiene, can support plaque control and limit the rise in gingival inflammation expressed as bleeding on probing during the first 12 weeks of fixed orthodontic treatment in adolescents.

This single-centre, randomised, assessor-blinded, controlled clinical study enrolled 41 adolescents aged 15–18 years referred from public dental services in the Lund region of Sweden for initiating fixed orthodontic treatment. Inclusion required good baseline oral hygiene and the absence of periodontal disease, defined as bleeding on probing below 10% [9]. Exclusion criteria included the presence of any chronic disease, use of antibiotics three months before or during the study, use of antiseptic mouthwashes during the study, and pregnancy or lactation.

The study protocol was approved by the Ethics Committee of the Swedish Ethical Review Authority (Dnr 2023-02385-01) and adhered to the Declaration of Helsinki and ISO 14155 [27] guidelines for good clinical practice. Written informed consent was obtained from all participants before enrolment, and, as per the local guidelines, their caregivers were informed about the study. No patients or members of the public were involved in the design, conduct, or reporting of the study.

After baseline measurements and individualised oral hygiene instruction, fixed orthodontic therapy was initiated. Participants were randomised 1:1 to a Control group (standard hygiene only) or a Test group (standard hygiene plus the home-use dual-light aPDT device).

Randomisation was performed after the baseline examinations. The allocation sequence was generated manually: the independent study monitor prepared envelopes containing the group allocations and shuffled them by hand to randomise their order, after which they were numbered sequentially. No block randomisation or stratification was applied. The resulting sequentially numbered, sealed, opaque envelopes ensured that enrolling investigators could not foresee the upcoming allocation. The CONSORT 2025 participant flow, including enrolment, allocation, follow-up, and analysis at each stage, is presented in the supplementary flowchart (Supplementary Figure S1). No formal a priori sample size calculation was performed for this trial. The study was designed to generate preliminary effect estimates within a single-centre setting and to inform the design and sample size calculations of future, adequately powered trials. As such, the study was not powered to detect statistically significant differences in participant-level mean outcomes, and the absence of significance at this level should not be interpreted as evidence of no effect, particularly given the consistent direction of effect observed across all three independent indices (VPI, BOP, and OPI) and the statistically significant site-level findings.

### 2.1. Intervention and Comparator

Standard hygiene, applied in both groups, included professional polishing at baseline and detailed home-care instructions following a standardised prophylactic protocol for fixed orthodontic treatment.

In addition, Test-group participants were trained in home use of the dual-light aPDT device. They were instructed to use the device five to seven times per week and to keep a diary of compliance. Each session consisted of rinsing the mouth with an indocyanine green-based photosensitiser solution, followed by 10 min of dual-light exposure, after which the teeth were brushed as instructed.

**Figure 1:**
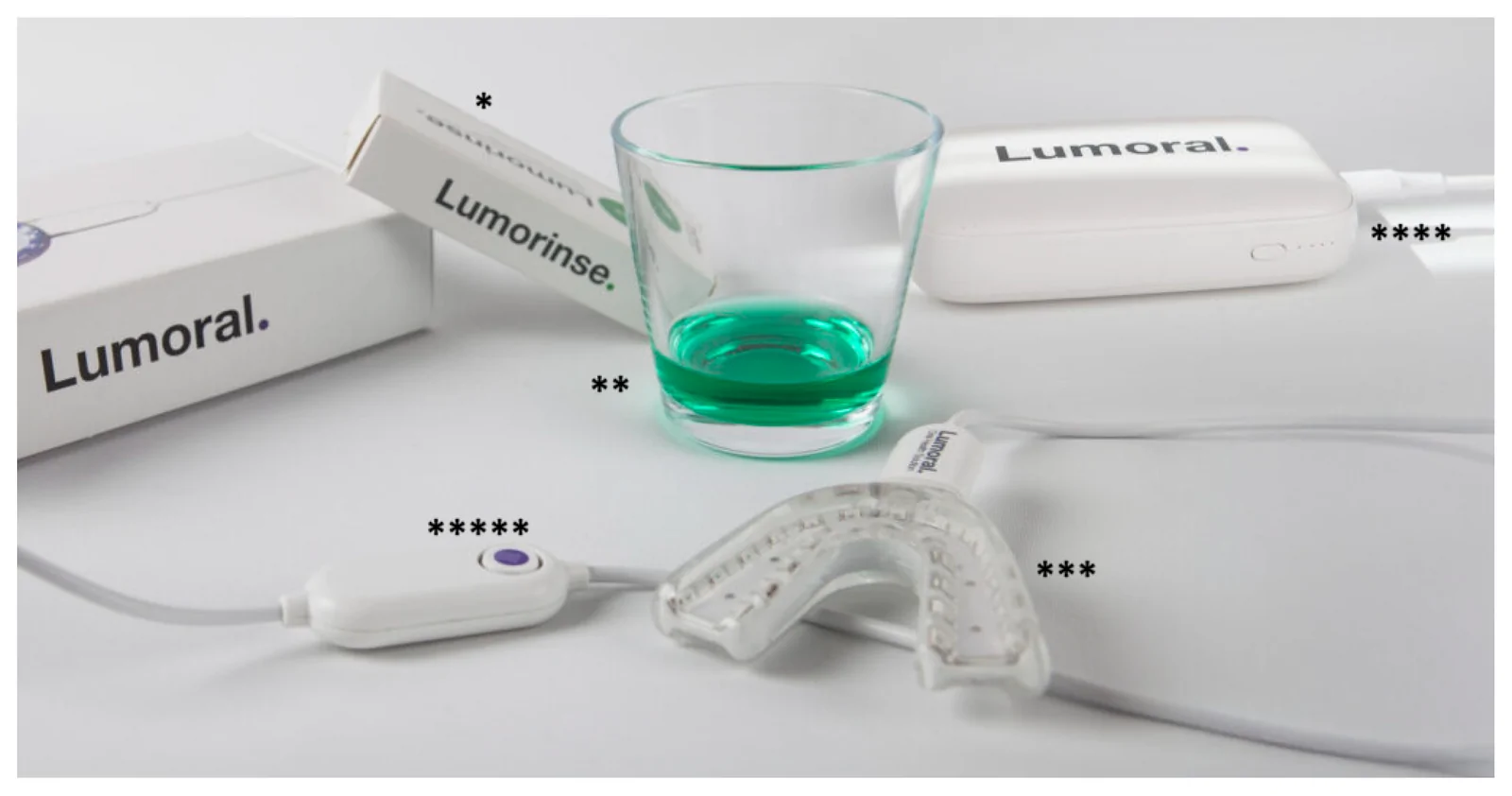
Lumoral^®^ Treatment dual-light aPDT device and accessories. The Lumorinse^®^ mouthrinse is provided in an effervescent tablet form (*), to be dissolved in 30 mL of water (**) using a supplied measuring cup. The mouthpiece (***) is connected to the power supply (****) via a cord, and activated (*****) to deliver simultaneous light to both the maxillary and mandibular dental arches. The device turns off automatically after ten minutes of exposure. Each LED component emits 405 nm and 810 nm light wavelengths simultaneously. Note: the glass shown in the photograph is for visual reference and is not provided with the device.

The dual-light aPDT device used in this study (Lumoral^®^ Treatment, Koite Health Ltd., Espoo, Finland) is a CE-marked home-use antibacterial device for the prevention and adjunctive treatment of bacterially driven oral diseases. It is used together with a CE-marked indocyanine green (ICG) photosensitiser mouthrinse (Lumorinse^®^, Koite Health Ltd.), which adheres to the dental biofilm (Figure 1).

The device provides dual-light action: 810 nm light activates ICG bound to the biofilm, while 405 nm antibacterial blue light is absorbed by intrinsic bacterial chromophores (mainly porphyrins and flavins). Combined, these wavelengths produce a sustained antibacterial effect [28,29,30]. When used alongside mechanical cleaning, the device acts on supragingival plaque and reduces the pathogenic biofilm burden on periodontal tissues [24,25,26]. The same wavelengths may also exert a photobiomodulatory effect on host tissues [31,32].

### 2.2. Outcome Measures and Timeline

The primary outcome was the Visible Plaque Index (VPI%). Secondary outcomes were Bleeding on Probing (BOP%) and the Orthodontic Plaque Index (OPI).

Measurements were taken at baseline and at 12 weeks using standardised indices. VPI% and BOP% were recorded at six sites per tooth on all teeth. OPI was evaluated from standardised intra-oral photographs taken at the 12-week visit.

VPI% was recorded as the percentage of tooth surfaces exhibiting visible plaque at six sites per tooth, in accordance with the Plaque Control Record [33]. BOP% was recorded as the percentage of sites exhibiting bleeding within 15 s of gentle probing, in accordance with standard periodontal assessment methodology [34]. OPI was assessed from standardised intra-oral digital photographs in accordance with the original index methodology [35]. Photographic assessment offers the additional advantage that images can be reviewed and confirmed by other investigators, enhancing reproducibility and transparency of the outcome assessment.

The VPI thresholds of 20% and 30% were selected a priori as clinically meaningful benchmarks for good oral hygiene in orthodontic patients. The 20% threshold reflects plaque levels long considered indicative of satisfactory plaque control in periodontal practice, based on the Plaque Control Record [33] and its subsequent use in periodontal maintenance and risk assessment [36]. The selected thresholds also reflect the broader principle that plaque accumulation must remain below a critical level to limit the risk of gingival and periodontal inflammation [37]. Given the relatively high baseline plaque levels observed in the present cohort, an additional threshold of 30% was prespecified to represent a clinically meaningful improvement while remaining a realistic treatment target for orthodontic patients. The proportion of participants achieving these thresholds was used as a complementary measure of clinically relevant hygiene performance, alongside the continuous VPI% outcome.

In addition to participant-level VPI% and BOP%, plaque- and bleeding-scoring points were calculated as a site-level outcome measure. For each participant, presence or absence of visible plaque and bleeding on probing was recorded dichotomously (1 = present, 0 = absent) at six sites per tooth (mesio-buccal, buccal, disto-buccal, mesio-lingual, lingual, disto-lingual) at both baseline and 12 weeks. These site-level scores were aggregated across all participants within each group to calculate the total proportion of plaque-positive and bleeding-positive sites at each timepoint. This approach was included because participant-level percentage indices, such as VPI% and BOP%, are derived as an average of averages across all sites within a participant, which may mask underlying changes occurring at a subset of sites. The site-level scoring approach allows for a more granular and sensitive assessment of change, since every individual measurement point across the full participant cohort contributes directly to the analysis, without the smoothing effect introduced by within-participant averaging. This level of measurement-point granularity is particularly valuable in a study with a modest sample size, where participant-level averaging alone may obscure clinically relevant changes that are nonetheless detectable when all measurement points are considered.

Participants were not given standardised instructions to either clean their teeth or refrain from cleaning before study visits, and no dietary restrictions were imposed on the day of a visit. Visits could occur at any time between 08:00 and 16:00.

### 2.3. Blinding

Study visits were performed by two qualified dentists (A.-M.R.J. and K.E.). All clinical assessments were conducted by a blinded assessor (A.-M.R.J.), a specialist in periodontology, while the unblinded investigator (K.E.) was responsible for participant allocation and device instruction. Clinical measurements were performed in a separate room from device handling; the blinded assessor used non-coded, unlabelled case report forms, and group allocation was not discussed with participants in the assessor’s presence. Data analysis was also performed blinded to allocation.

### 2.4. Monitoring

An external clinical research associate, independent of the investigative team, monitored study conduct. Monitoring ensured adherence to ISO 14155:2020 [27] Good Clinical Practice standards and included verification of case report forms against source data. Clinical data were inspected for discrepancies before analysis as part of the monitoring procedures.

### 2.5. Statistical Analysis

Continuous variables are summarised as means with standard error of the mean (SEM) or as medians with ranges, as appropriate. Within-group changes from baseline to 12 weeks were analysed with paired *t*-tests or Wilcoxon signed-rank tests, depending on the distribution. Between-group comparisons used unpaired *t*-tests or Mann–Whitney U tests. Between-group comparisons of plaque-positive and bleeding-positive sites were made using the chi-square test. A two-sided *p*-value < 0.05 was considered statistically significant. Analyses were performed with GraphPad Prism (version 10.4.2; GraphPad Software, San Diego, CA, USA). The final analysis was conducted on a per-protocol basis, including all participants who completed baseline and 12-week assessments according to their randomised group allocation. It should be noted that the site-level chi-square analyses do not account for the clustering of observations within patients; these results are therefore considered exploratory.

## 3. Results

### 3.1. Baseline Characteristics

**Figure 2:**
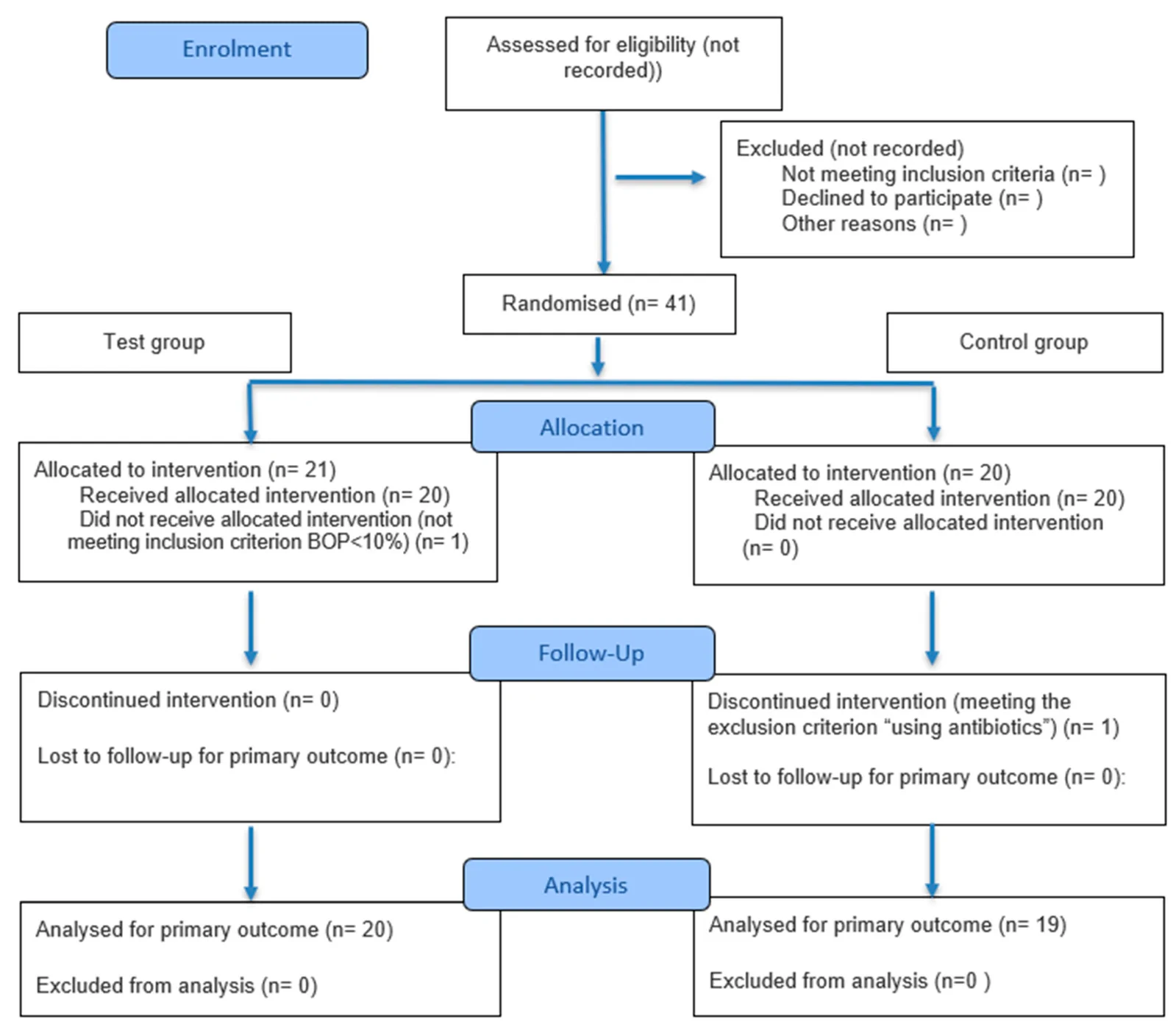
CONSORT flow chart.

All participants were 15–17 years of age. No significant baseline differences were observed between groups in demographic variables. No participant reported a chronic illness or regular medication use. In the Test group, one participant was excluded because baseline BOP exceeded the 10% inclusion threshold and therefore did not receive the allocated intervention. Thus, of the 41 randomised participants (21 Test, 20 Control), one participant per arm did not continue, leaving 20 Test and 19 Control participants in the analysis. See Figure 2.

In total, 39 participants’ data were included in the analysis: 20 in the Test group and 19 in the Control group. The final analysis was conducted on a per-protocol basis, including all participants who completed baseline and 12-week assessments according to their randomised group allocation.

Table 1 summarises sex distribution and self-reported tobacco use by group.

### 3.2. Compliance and Safety

Test-group participants were asked to keep a diary of device use. Twelve of the 20 participants (60%) returned their diaries at or after the 12-week visit. Among the 60% of participants who returned their diaries, mean self-reported compliance was 79%, corresponding to approximately 5.5 days of use per week; this figure should be interpreted as an estimate derived from diary returners rather than the full Test group. The 40% non-return rate is acknowledged as a limitation of the compliance estimate.

No device- or protocol-related adverse events were reported during the study. Two minor incidents with the dual-light aPDT devices were a broken mouthpiece (one participant) and a broken power supply (one participant), both of which were instantly replaced with no major pauses in device use.

### 3.3. Visible Plaque Index (VPI)

In the Test group, the mean ± SEM VPI% was 55.0% ± 5.93%, and 58.2% ± 6.22% in the Control group at baseline, with no statistically significant difference between groups (*p* = 0.844). At 12 weeks, mean ± SEM VPI% had decreased to 48.0% ± 5.09% in the Test group and to 54.4% ± 5.71% in the Control group, again without a statistically significant between-group difference (*p* = 0.483). The mean within-group change in VPI% was −7.0% in the Test group and −3.7% in the Control group.

**Figure 3:**
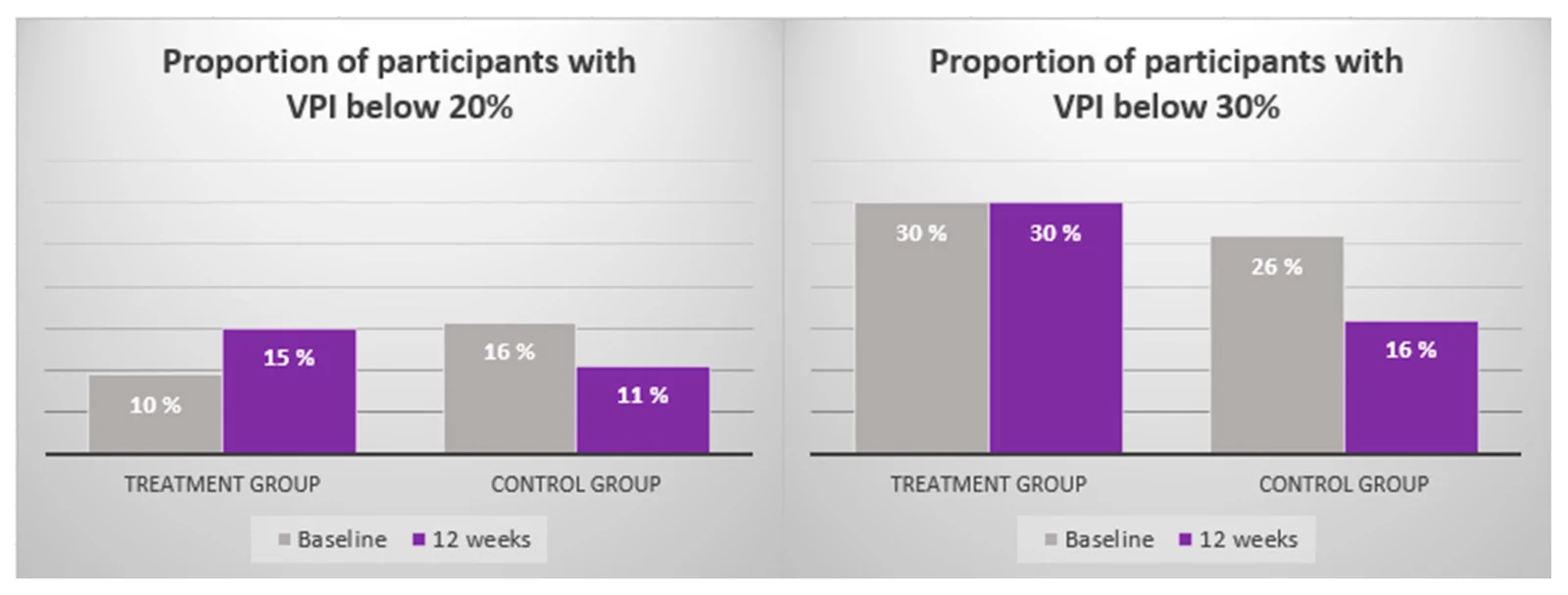
Proportion of participants with VPI below 20% (**left**) and below 30% (**right**) at baseline and at 12 weeks. VPI, visual plaque index.

At baseline, the proportion of participants with VPI below 20% was 10% in the Test group and 16% in the Control group, respectively, and the proportion of participants with VPI below 30% was 30% in the Test group and 26% in the Control group, respectively. At the 12-week visit, the proportion of participants with VPI below 20% increased to 15% in the Test group and decreased to 11% in the Control group, respectively, and the proportion of participants with VPI below 30% remained at 30% in the Test group and decreased to 16% in the Control group, respectively. None of these between-group differences reached statistical significance. See Figure 3.

### 3.4. Plaque Scoring Points

Plaque scoring was performed as a dichotomous assessment at six sites per tooth (mesio-buccal, buccal, disto-buccal, mesio-lingual, lingual, disto-lingual) for the presence or absence of visible plaque at both baseline and 12-week visits. A score of 1 was recorded when visible plaque was present (positive plaque score), and 0 when absent (negative plaque score). In the Test group (*n* = 19; one participant’s data missing), this resulted in a total of 3440 plaque scoring points, and in the Control group (*n* = 18; one participant’s data missing), a total of 3268 plaque scoring points. At baseline, the total positive plaque score was 2115/3440 (61.5%) in the Test group and 2116/3268 (64.7%) in the Control group. At 12 weeks, the corresponding values were 1780/3440 (51.7%) and 1915/3268 (58.6%), respectively—a reduction of 335 positive sites (−15.8%) in the Test group and 201 positive sites (−9.5%) in the Control group. The between-group difference was statistically significant at both timelines (Table 2).

### 3.5. Bleeding on Probing (BOP%)

In the Test group, the mean ± SEM BOP% was 4.8% ± 0.5% at baseline and 7.2% ± 1.7% at 12 weeks; in the Control group, the corresponding values were 4.7% ± 0.6% and 10.4% ± 2.0%, with no statistically significant between-group differences (*p* = 0.735 at baseline and *p* = 0.081 at 12 weeks). The mean within-group increase in BOP% was 2.4% in the Test group and 5.7% in the Control group.

**Figure 4:**
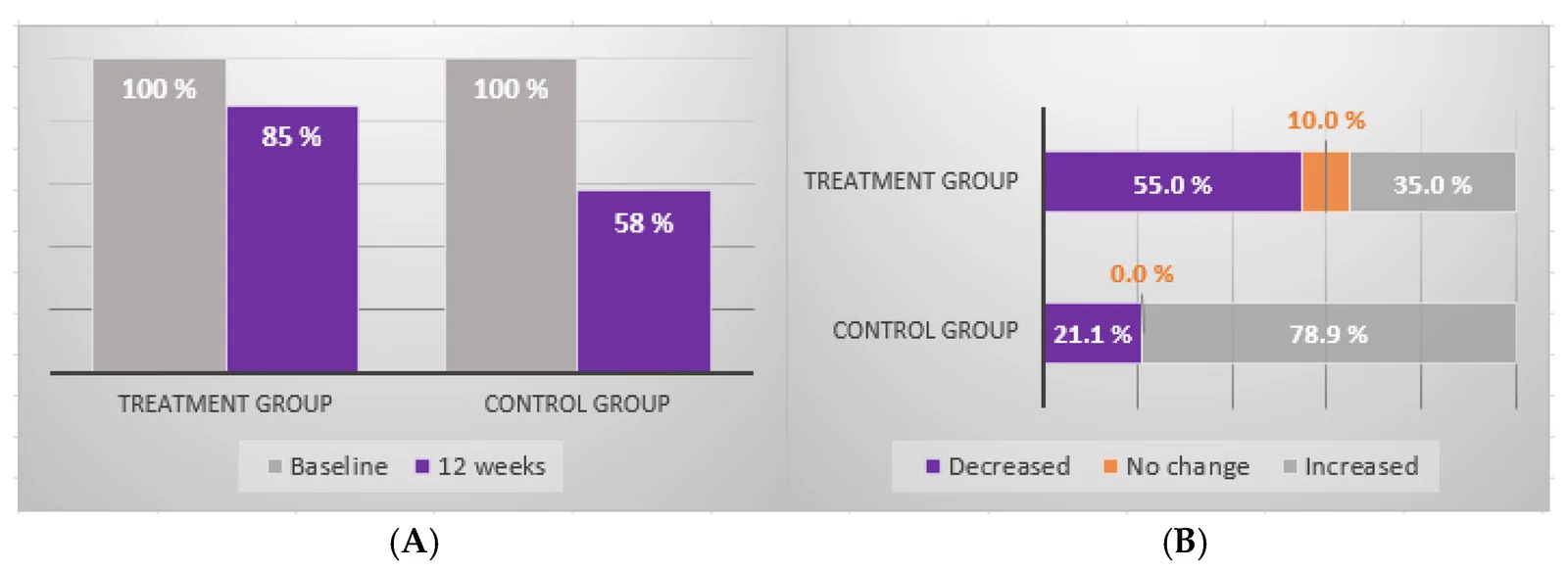
(**A**): Proportion of participants with BOP below 10% at baseline and at 12 weeks. (**B**): Proportion of participants with either a decrease, no change, or an increase in BOP% compared from baseline to the 12-week timepoint. BOP, bleeding on probing.

The proportion of participants with BOP ≤ 10% was 100% in both groups at baseline. At 12 weeks, 85% (17/20) of the participants in the Test group had maintained their BOP at ≤10%, compared to 58% (11/19) in the Control group. See Figure 4A.

Overall BOP% decreased between baseline and 12 weeks in 55.0% (*n* = 11) of participants in the Test group, versus 21.1% (*n* = 4) in the Control group. BOP% increased in 35.0% (*n* = 7) of participants in the Test group, versus 78.9% (n = 15) in the Control group. Two Test-group participants (10.0%) showed no change in BOP%. See Figure 4B.

### 3.6. Bleeding Scoring Points

Bleeding on gums was assessed dichotomously at six sites per tooth (mesio-buccal, buccal, disto-buccal, mesio-lingual, lingual, disto-lingual) for the presence of bleeding on probing at both baseline and 12-week visits. A score of 1 was recorded when bleeding occurred (positive bleeding score), and 0 when absent (negative bleeding score). In the Test group, a total of 3440 bleeding-scoring points were available, and in the Control group, 3268. At baseline, the total positive bleeding score was 201/3440 (5.8%) in the Test group and 165/3268 (5.0%) in the Control group. At 12 weeks, the corresponding values were 238/3440 (6.9%) and 378/3268 (11.6%), respectively. The overall change from baseline to 12 weeks was +18.4% in the Test group and +129.1% in the Control group. The between-group difference was not statistically significant at baseline (*p* = 0.152), but was at the 12-week timepoint (*p* < 0.001) (Table 3).

### 3.7. Orthodontic Plaque Index (OPI)

**Figure 5:**
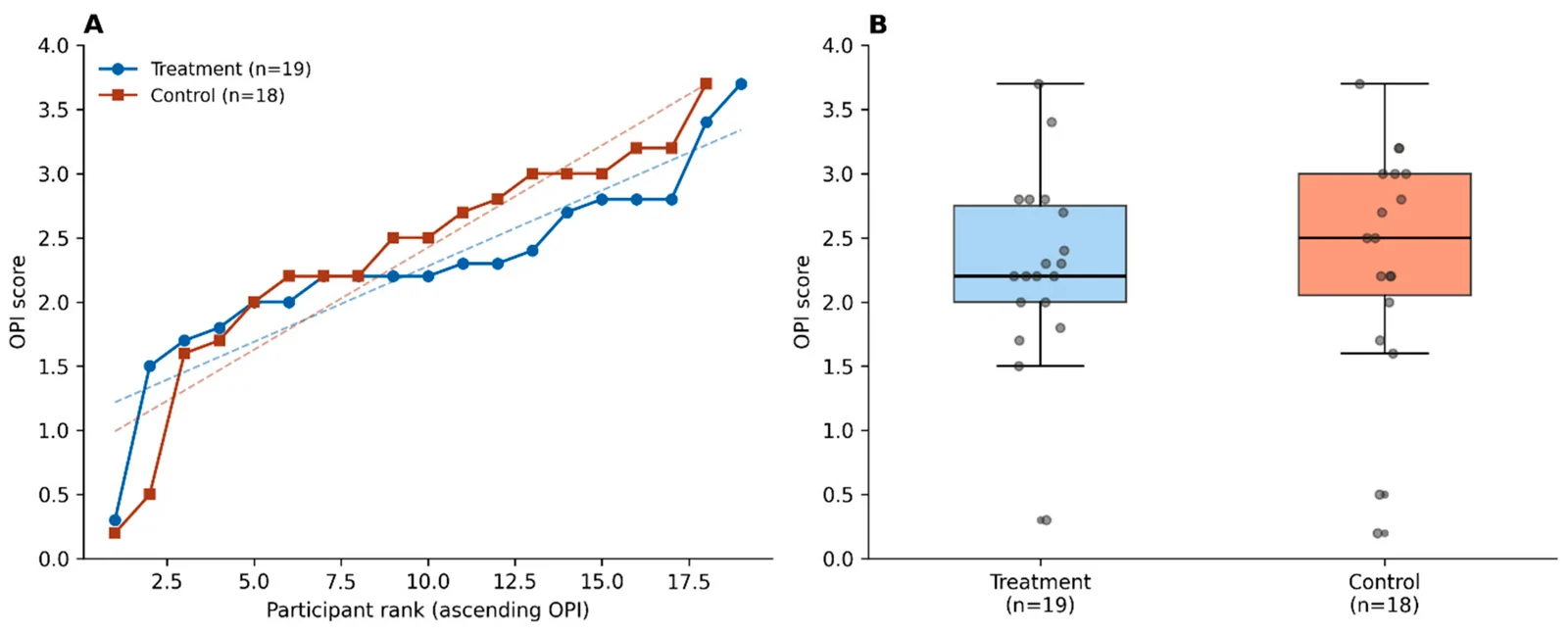
(**A**) Per-participant OPI values plotted in ascending order with a linear trend line for each group. (**B**) Boxplot showing the median, interquartile range, and full range of OPI values for the Treatment and Control groups at 12 weeks, with individual data points overlaid. OPI, orthodontic plaque index.

In the Test group (*n* = 19; one participant’s photographs were missing), average OPI values ranged from 0.3 to 3.7 (median 2.2). In the Control group (*n* = 18; one participant’s photographs were missing), values ranged from 0.2 to 3.7 (median 2.5). Figure 5A plots the per-participant OPI values for each group in ascending order, while Figure 5B presents the same data as a boxplot to illustrate the distribution and variability between groups.

## 4. Discussion

This randomised, assessor-blinded controlled trial evaluated whether regular home use of dual-light antibacterial photodynamic therapy (aPDT) supports oral hygiene during fixed orthodontic treatment in adolescents. Across all three indices—VPI, BOP, and OPI—outcomes consistently favoured the Test group, with the most marked between-group differences seen at the site level, though these analyses should be interpreted cautiously given the clustering of measurement sites within patients. Together, these findings suggest that adjunctive dual-light aPDT may help compensate for the well-known limitations of mechanical plaque removal around fixed appliances.

VPI% decreased in both groups over 12 weeks, indicating that oral hygiene did not deteriorate during the early phase of fixed orthodontic treatment. This is itself notable, as fixed appliances are typically associated with increased plaque retention. The larger mean reduction in the Test group, together with a lower proportion of plaque-positive sites at 12 weeks in an exploratory site-level analysis (*p* < 0.001), supports a role for dual-light aPDT as an adjunct to mechanical hygiene in this setting.

It is well established that gingival bleeding typically increases during fixed orthodontic treatment because of plaque retention and the difficulty of maintaining oral hygiene around brackets [2,4,38]. In line with this, mean BOP% increased in both groups, but the rise was smaller in the Test group; more participants in that group maintained BOP ≤ 10% (85% vs. 58%), and the proportion of bleeding-positive sites was lower in the Test group at 12 weeks in an exploratory site-level analysis (*p* < 0.001). These convergent signals suggest that adjunctive aPDT may attenuate the gingival inflammatory response that typically accompanies fixed appliances, even in adolescents with low baseline BOP.

The Orthodontic Plaque Index (OPI), based on blinded photographic assessment of plaque around brackets, points in the same direction. The Test group showed a flatter distribution of OPI scores and a lower median than the Control group, although the between-group difference did not reach statistical significance, in keeping with the limited sample size and the inter-individual variability typical of bracket-level plaque measurements. These trends are consistent with the magnitude and direction of effects reported in earlier randomised studies of dual-light aPDT in peri-implant and periodontal disease [24,25].

The convergent pattern across plaque and bleeding endpoints is unlikely to be explained by changes in plaque quantity alone and may reflect changes in biofilm composition. Dental plaque is a highly structured microbial ecosystem whose pathogenicity is determined not only by its mass but also by its microbial profile and metabolic activity [39,40,41]. Fixed orthodontic appliances induce ecological shifts toward a more anaerobic and periodontopathogenic microbiota, even when overall plaque levels do not change substantially [4,5]. Recent systematic evidence supports that orthodontic treatment is associated with an increased prevalence of periodontal pathogens and oral dysbiosis, particularly with fixed appliances [42,43].

Antibacterial photodynamic therapy (aPDT) exerts its effect through the generation of reactive oxygen species, leading to oxidative damage of bacterial cell membranes and disruption of biofilm integrity [44,45]. In addition to its broad-spectrum antimicrobial activity, repeated dual-light aPDT reduces bacterial load and inhibits adaptive responses within oral biofilms without selecting for antimicrobial resistance [28,29,30].

This mechanism may help explain why the trend toward less gingival inflammation in the Test group was relatively larger than the difference in mean VPI%. By selectively suppressing pathogenic species within the biofilm, aPDT could reduce the inflammatory burden at the gingival level even when overall visible plaque is not dramatically reduced. This interpretation is consistent with microbiome research emphasising that qualitative dysbiosis—rather than absolute biofilm quantity—is a key driver of periodontal inflammation [41,42]. The present study did not include microbiological sampling, so this remains a hypothesis to be tested directly in future work.

The results align with earlier observations that repeated dual-light aPDT, combining 405 nm and 810 nm wavelengths, provides a sustained antibacterial effect on plaque biofilms without systemic antibiotics or chlorhexidine-type antiseptics [24,25,46]. The home-use format addresses a historical limitation of aPDT—its inaccessibility outside the clinic—and the method has a favourable safety profile in repeated daily use [25].

Adolescents are a population in whom optimal oral hygiene is difficult to maintain because of behavioural variability and the physical challenges posed by fixed appliances [4,9]. An easy-to-use, home-based adjunct that supports biofilm control without compromising safety is, therefore, clinically attractive. The present study demonstrates feasibility, acceptable safety, and consistent direction-of-effect across three independent indices over 12 weeks in this group.

Taken together, the present findings align with previous peri-implant, plaque, and periodontitis studies and reinforce the anti-inflammatory and plaque-controlling potential of dual-light aPDT [24,25,26,46]. Adolescents undergoing fixed orthodontic treatment may particularly benefit from this adjunct, which can help compensate for the limitations of mechanical plaque removal during this vulnerable treatment period.

Several limitations should be acknowledged. The trial was conducted at a single centre with a modest sample size. No formal a priori sample size calculation was performed for this trial. The study was designed to generate preliminary effect estimates within a single-centre setting and to inform the design and sample size calculations of future, adequately powered trials. As such, the study was not powered to detect statistically significant differences in participant-level mean outcomes, and the absence of significance at this level should not be interpreted as evidence of no effect, particularly given the consistent direction of effect observed across all three independent indices (VPI, BOP, and OPI) and the statistically significant site-level findings. Furthermore, the site-level chi-square analyses do not account for the clustering of multiple measurement sites within the same patient; this may result in underestimated standard errors and inflated statistical significance for the site-level findings, which should therefore be interpreted as exploratory and supportive rather than confirmatory. One participant in each group did not complete the study—a Control-group participant who discontinued because of systemic antibiotic treatment and a Test-group participant excluded for not meeting the baseline BOP inclusion criterion. As the analysis was conducted on a per-protocol basis, these two participants were not included; this departure from a strict intention-to-treat approach should be taken into consideration when interpreting the results. Compliance with the home device was self-reported via diary, with diaries returned by 60% (12/20) of Test-group participants, so the 79% mean compliance figure should be interpreted as an estimate from returners rather than the full Test group. As treatment effectiveness is plausibly related to actual device usage, the true level of adherence across the full Test group remains uncertain, and this should be considered when interpreting the magnitude of the observed treatment effect. Additionally, the between-group difference in BOP% at 12 weeks approached statistical significance (*p* = 0.081), which, given the modest sample size, suggests a trend favouring the Test group. A Hawthorne effect, arising from participation in a clinical trial where the participants are not blinded, may also have contributed to the within-group plaque reductions observed in both arms. Pre-screening data were not collected systematically, which prevents complete reporting against the CONSORT 2025 framework. Microbiological sampling was not performed, so the proposed mechanism via shifts in biofilm composition remains hypothesis-generating. Strengths of the study include continuous independent monitoring under ISO 14155, a periodontology specialist as blinded assessor, and consistent direction-of-effect across three independent indices including site-level analyses.

As oral healthcare continues to seek sustainable, evidence-based innovations for patient self-care, adjunctive aPDT merits further evaluation, particularly in populations prone to plaque accumulation and gingival inflammation due to anatomical or behavioural constraints. The consistency of effect direction across VPI, BOP, and OPI in this study, together with concordance with earlier dual-light aPDT studies, provides a rationale for adequately powered, multicentre trials in larger and more diverse orthodontic populations, ideally with longer follow-up and microbiological endpoints.

## 5. Conclusions

In adolescents undergoing fixed orthodontic treatment, regular home use of dual-light aPDT was safe and was associated with better site-level plaque control and a smaller rise in gingival bleeding at the site level than standard hygiene alone over 12 weeks. Although the study was not powered to detect small participant-level differences, the consistent direction of effect across three independent indices supports further evaluation of dual-light aPDT as an adjunct to mechanical hygiene during this vulnerable treatment period. Clinically, adjunctive home-use dual-light aPDT may help compensate for the limitations of mechanical plaque removal around fixed orthodontic appliances, particularly in adolescents in whom maintaining hygiene during treatment is challenging. These findings should be regarded as preliminary given the modest sample size of this single-centre study, and larger, adequately powered multicentre trials are needed before definitive clinical recommendations regarding dual-light aPDT as an adjunct to orthodontic hygiene can be made.

## Figures and Tables

**Table 1:** Participant gender and self-reported use of tobacco products, and BOP% and VPI% measurements at baseline within groups. Between-group differences were assessed using the chi-square test for sex distribution, Fisher’s exact test for tobacco use, and the Mann–Whitney U test for BOP% and VPI%. No statistically significant between-group differences were observed at baseline. Abbreviations: BOP, bleeding on probing; VPI, visible plaque index; SEM, standard error of the mean.

	Gender		Use of Tobacco Products		Baseline Clinical Measurements (Mean ± SEM)	
	**Female**	**Male**	**No**	**Yes**	**VPI%**	**BOP%**
Treatment group, *n* = 20	11	9	19	1	55.0% ± 5.93%	4.8% ± 0.5%
Control group, *n* = 19	12	7	18	1	58.2% ± 6.22%	4.7% ± 0.6%
Between-group differences	*p* = 0.605		*p* = 1.000		*p* = 0.844	*p* = 0.735

**Table 2:** Changes in positive plaque scores within groups. Between-group comparisons were performed using the chi-square test.

	Baseline Plaque Scores	12-Week Plaque Scores	Change from Baseline to 12 Weeks	Change % from Baseline to 12 Weeks	Between-Group Differences (Baseline; 12 Weeks)
Test group	2115	1780	−335	−15.8%	*p* = 0.006; *p* < 0.001
Control group	2116	1915	−201	−9.5%	

**Table 3:** Changes in positive bleeding scores within groups. Between-group comparisons were performed using the chi-square test.

	Baseline Bleeding Score	12-Week Bleeding Score	Change from Baseline to 12 Weeks	Change % from Baseline to 12 Weeks	Between-Group Differences (Baseline; 12 Weeks)
Test group	201	238	+37	+18.4%	*p* = 0.152; *p* < 0.001
Control group	165	378	+213	+129.1%	

## Data Availability

The datasets presented in this article are subject to the European General Data Protection Regulation (EU) 2016/679 and are not readily available because they may include pseudonymized personal data. Requests to access the datasets should be directed to the corresponding author. The secure transmission of data sets may be delayed due to the anonymization process.

## Supplementary Materials

The following supporting information can be downloaded at: https://www.mdpi.com/article/10.3390/dj14090564/s1, Table S1: CONSORT 2025 Consort checklist [47].

## Abbreviations

The following abbreviations are used in this manuscript:

aPDT	antibacterial photodynamic therapy
VPI	Visible Plaque Index
BOP	Bleeding on Probing
OPI	Orthodontic Plaque Index
